# Event and Comment

**Published:** 1920-05

**Authors:** 


### DENTAL REGISTER

THE MAGAZINE OF DENTISTRY

Vol. LXXIV MAY, 1920 No. 5

### EVENT AND COMMENT

**Government Tax on Dental Supplies** Two bills of very considerable dental interest are before Congress at this time, and we are printing them in our editorial space with the hope that they may be given proper discussion in our profession, and that proper congressional action shall be had.

It would seem almost incredible that Congress, or anyone else who gives any thought to the importance of dental and medical health, would even suggest the propriety of placing anything in the way of prohibiting the good health of the people. No one who will stop a moment to think of the therapeutic value of gold in the surgical art will be willing to deny suffering humanity the free and uninterrupted use of this most valuable method of saving the teeth from the most universal disease known to the world. Everybody sooner or later suffers from decayed teeth, and many people suffer to the limit of their lives. It does not seem possible that we can have no more gold or platinum now. These are the metals that at the present time form the basis of our therapeutic art. Of course some of us will be able, possibly, to get the gold we need if we have the money to buy it with, but unfortunately the people who need it most have not an abundance of money with which to purchase. If our government can not collect the needed tax money in some other way, we shall certainly have to pay a larger cost in a more costly form of degenerated humanity. Surely there must be a better way out than this. And then on top of this comes the proposal that our artificial teeth must be taxed also. Now, "can you beat it?"—in the language of the street. Think of it, you poor, old, toothless derelicts. The only comfort we can see is that as time goes on we shall have no use for false teeth anyway, since there will be nothing worth while chewing anyway. Our legislators are no doubt doing all they can think of to help out in these trying times and we want to help all we can, but we can't help saying some things that won't look nice in print, such as !—!—x—x—?—?—*—*—!—?—x—?—!

---

### A PROPOSED EXCISE TAX OF $10 PER OUNCE ON GOLD

Congressman Louis T. McFadden, of Canton, Pa., recently introduced in Congress a bill known as H. R. 13200, providing for an excise tax of $10 per ounce on all gold, except that used for monetary purposes and for some limited dental purposes, such as governmental and free clinic uses, and also for corrective and restorative dental work for children under 15 years.

This legislation is sponsored by the American Mining Congress and is supposed to be approved, if not strongly supported, by the banking interests of the country. Its alleged purpose is to stimulate an increased gold production, and there may be some very good arguments for offering some additional incentive to the gold mining industry, in order to produce the required amount of this very important and necessary metal. This tax is to be distributed as a premium or a bonus to the producers of new gold on a basis of $10 for each ounce mined. However, unless all gold used in any way to correct physical defects is exempted from the provisions of this bill, the destructive results—both from a humanitarian and an economic standpoint—will be so far-reaching as to more than counterbalance any constructive benefits wherein economies and the mining industry are considered.

The teeth rank first and the eyes second in any classification of physical defects. The importance and necessity of correcting defective vision has long been recognized, but only in recent years has the importance of neglected mouth conditions, as a positive contributing factor in disease, become generally recognized. At the present time many industrial institutions are recognizing the individual unit as an asset and have established both medical and dental clinics to conserve health, promote efficiency, and increase production. Therefore, if Congress should enact legislation which will in any way tend to restrict the benefits accruing from the correction of such dental and eye defects the far-reaching influence, in the final analysis, will be destructive rather than constructive. The correction of these defects is fundamentally a constructive service and can not in any sense be considered as a luxury. Thus the best interest of society, in its broadest sense, will be served by recognizing the merits of such exemptions. This will also tend to stimulate an increased interest in health conservation in general.

Another very objectionable bill is pending in Congress, introduced by Congressman Isaac Bacharach, of Atlantic City, N. J., and known as H. R. 7785. This bill provides for a greatly increased duty on surgical and dental instruments and artificial teeth, and it passed the House August 2, 1919. The Senate Finance Committee reported this to the Senate March 2, 1920, without amendments. All such added expenses become a fixed overhead and must ultimately be distributed to a long-suffering public, whose burdens are already sufficiently oppressive. Therefore, this becomes more than a dentist's problem, and protests from the public will be voiced when fully advised of the situation.

In view of the foregoing, I urgently request that each reader promptly write his Congressman, emphasizing the importance of exempting from the provisions of H. R. 13201 all gold used in any way to correct physical defects. Also promptly write his Senators, requesting them to oppose the increased duty on surgical and dental instruments and artificial teeth. Discuss this proposed legislation with your influential patients and secure their co-operation.

H. R. 13201 is before the Ways and Means Committee of the House of Representatives and I append hereto a full list of the personnel of said committee. It is especially important that these committeemen be communicated with and the officers of any dental organization will be fully justified in promptly speaking for their members, since time is an important factor.

WAYS AND MEANS COMMITTEE

Joseph W. Fordney, Chairman, Mich.; J. Hampton Moore, Pa.; William R. Green, Iowa; Nicholas Longworth, Ohio; Willis C. Hawley, Ore.; Allen T. Treadway, Mass.; Ira C. Copley, Ill.; Luther W. Mott, N. Y.; George M. Young, N. Dak.; James A. Frear, Wis.; John Tilson, Conn.; Isaac Bacharach, N. J.; Lindley H. Hadley, Wash.; Charles B. Timberlake, Colo.; George M. Bowers, W. Va.; Claude Kitchin, N. Car.; Henry T. Rainey, Ill.; Cordell Hull, Tenn.; John N. Garner, Texas; James W. Collier, Miss.; Clement C. Dickinson, Mo.; William A. Oldfield, Ark.; Charles R. Crisp, Ga.; John F. Carew, N. Y.; Whitmell P. Martin, La.

HOMER C. BROWN, D.D.S.,
Chairman Legislative Committee,
National Dental Association,
April 19, 1920. 609 Hartman Building.

---

### THE FORSYTH INFIRMARY REPORT

The following report of work done by the Forsyth Dental Infirmary for the year ending December, 1919, is sufficient evidence of the value of the enormous amount of work done in this institution. If there is any one who is not now willing to recognize the necessity for the further prosecution of this form of humanitarian endeavor, we would suggest that he spend his summer vacation inspecting the work that is now being accomplished in the Forsyth and Eastman infirmaries. We would also recommend that when he has made this inspection to his satisfaction that he complete the visit by a trip to a few of the prominent dental offices in some of the larger cities where the extraction of teeth is practiced as a specialty for adult patients. Any one who will make such a tour of inspection will realize as never before what should be done to save the teeth of future patients by the more careful treatment of our children's teeth. Incidentally, we believe such a trip will forever silence the criticism that is now being directed against the training of women as dental hygienists. We believe just now she is the most effective agency we have for securing better health of the teeth for the rising generation. If we are to stop the present craze for extracting all suspiciously diseased teeth, we can not do better work for conservative dentistry than by introducing better methods for saving by every means the teeth of the younger children. "Juvenile dentistry by prophylactic efforts of an approved character" should be the slogan for general as well as dental health.

This report should receive the most thoughtful consideration of every dentist, as it furnishes many valuable suggestions for improvement in private practice that some of our institutions have already put into effective use. We need more institutions and many more women who are willing to devote themselves to the education required in dental hygiene work. We also need more dental surgeons and dental and medical scientists who will study our problems and train our hygienists.

### SOME FORSYTH INFIRMARY STATISTICS

In the general clinic an average of 80 cases were treated each day. In a clinic, with 16 regular operators each day, 24,000 patients were treated last year. In the general clinic a total of 154,027 operations were made; 111,000 fillings were made; 24,000 root-canal treatments were made; 18 prophylactic treatments were made; nearly 700 operations for adenoids and tonsils were made; nearly 200 cases of orthodontia were treated. This large clinic will undoubtedly be materially increased in next report.

The following detailed report of the extracting clinic is interesting and gives a fair report of the character and great need of this work:

*Extracting Clinic*—Number of patients, 14,447; mouthwash cases, 2,464; N20 administrations, 115; Novocaine administrations, 5,927; ethyl chloride administrations, 8,904; total number of teeth extracted, 40,887; temporary teeth extracted, 33,947; sixth-year molars extracted, 5,756; other extractions, 483; number of abscess cases, 40; oral surgery cases, 57; oral surgery treatments, 273.

The following schools contributed through their pupils to the work of the Forsyth Infirmary: Boston city schools, 12; parochial schools, 17; suburban schools, 14; institutions and societies, 37.

*Oral Hygiene Department*—Children examined, 23,881; visits made, 70,061; buttons awarded for clean teeth, 2,786. Twenty-nine schools were awarded "completed certificates."

In the Dental Hygiene Clinic the following clinic is made:

Hours in surgical clinic, 5,361; hours in dental clinic, 745; hours in extracting clinic, 3,126; hours in sterilizing room, 532; hours in oral hygiene room, 1,827; hours in research laboratory, 210; hours in prophylactic clinic, 4,000; number of prophylactic treatments, 3,397.

